# Human health risk assessment data of trace elements concentration in tap water—Abeokuta South, Nigeria

**DOI:** 10.1016/j.dib.2018.04.041

**Published:** 2018-04-19

**Authors:** Ogbiye S. Samuel, Emenike C. PraiseGod, Tenebe I. Theophilus, Kafi C. Omolola

**Affiliations:** a Department of Civil Engineering, Covenant University, Canaanland, Ota, Ogun State, Nigeria; b School of Water, Energy and Environment, Water Science Institute, Cranfield University, Bedfordshire, United Kingdom

## Abstract

Constant drinking water monitoring schemes are necessary because hazardous substances tend to enter water bodies through geodetic and anthropogenic sources. The main goal of this study was to evaluate the human health risk assessment posed by high fluoride and iron concentration in tap water used for domestic activities and consumption. In this study, the concentration of fluoride in tap water varied at different locations, ranging from 0.48 mg/L to 1.84 mg/L with an average value of 1.23 mg/L while that of iron ranged from 0.02 to 2.96 mg/L. The cluster analysis displayed three popular groups in which the samples can be classified. The non-carcinogenic risk was determined with defined methods outlined by US EPA considering dermal and ingestion pathways. Total Hazard Index greater than 0.8 for fluoride consumption in the analyzed locations was obtained from location R16, R17, R15, R4, and R6.

**Specifications table**

**Table:** 

Subject area	Water Resources and Environmental Engineering
More specific subject area	Water Quality and health-risk assessment
Type of data	Tables and figures
How data was acquired	Location visits, Field sampling collection, ionic concentration analysis using standard analytical procedure [1], flame absorption spectrophotometer (FAAS), potentiometric ion-selection electrode.
Data format	Filtered, analyzed
Experimental factors	Measuring the values of ionic and fluoride content of tap water samples. Calculating the human health risk assessment followed after the concentration of fluoride was obtained.
Experimental features	Determining the ionic concentration of major water quality parameters and some trace metals in tap water at the point located in the study map. All samples were stored according to standard procedures before analysis was carried out.
Data source location	Abeokuta South, Nigeria. 3.341 E – 3.386 E and 7.121 N – 7.192 N
Data accessibility	The data are available with this article

**Value of the data**

● High fluoride concentration in water can lead to severe health implications in humans. Therefore, monitoring toxicity levels in water is essential.
● Human health risk assessment is vital since fluoride can be adsorbed through several pathways.
● Children and deprived population should be the target of public health policymakers to ensure they receive proper sensitization and preventive programs that will improve their health status.
● The data will help provide proper monitoring initiative to curtail fluoride contamination in groundwater which would serve as an efficient mechanism to reduce fluoride intake.

## 1 Data

**Fig. 1:**
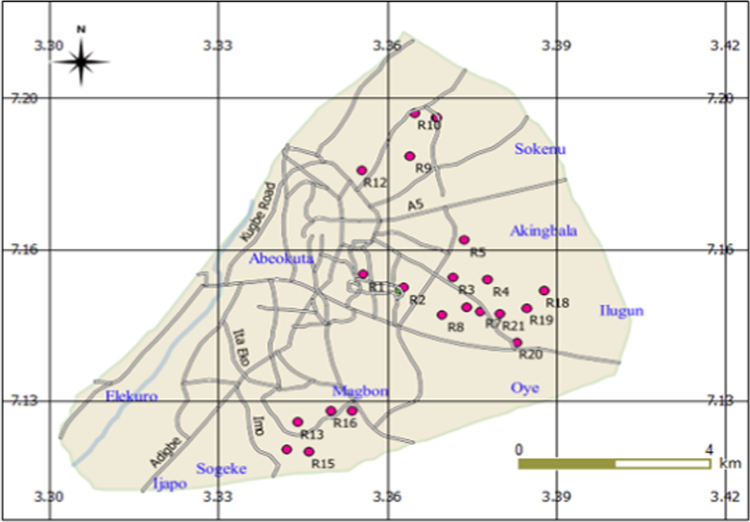
Map of study area indicating the sample locations.

In the data, tap water quality analysis was carried out in Abeokuta South in Ogun State, Nigeria at the locations in the map shown in Fig. 1. The region lies between latitude 7.17°N and 7.25°N and longitudes 3.28°E and 3.43°E with an estimated population of 451, 607 persons, growing at a rate of 3.5% yearly [2]. The data presented in this article provides information on classification of water samples and human health risk analysis associated with the intake of excess fluoride and iron concentration in water.

(*source:**[4]*).

## 2 Experimental design, materials, and methods

In this study, 21 locations were chosen for water analysis (Fig. 1). 63 tap water samples were collected, analyzed and compared with water quality standards outlined by the World Health Organization [3].

**Fig. 2:**
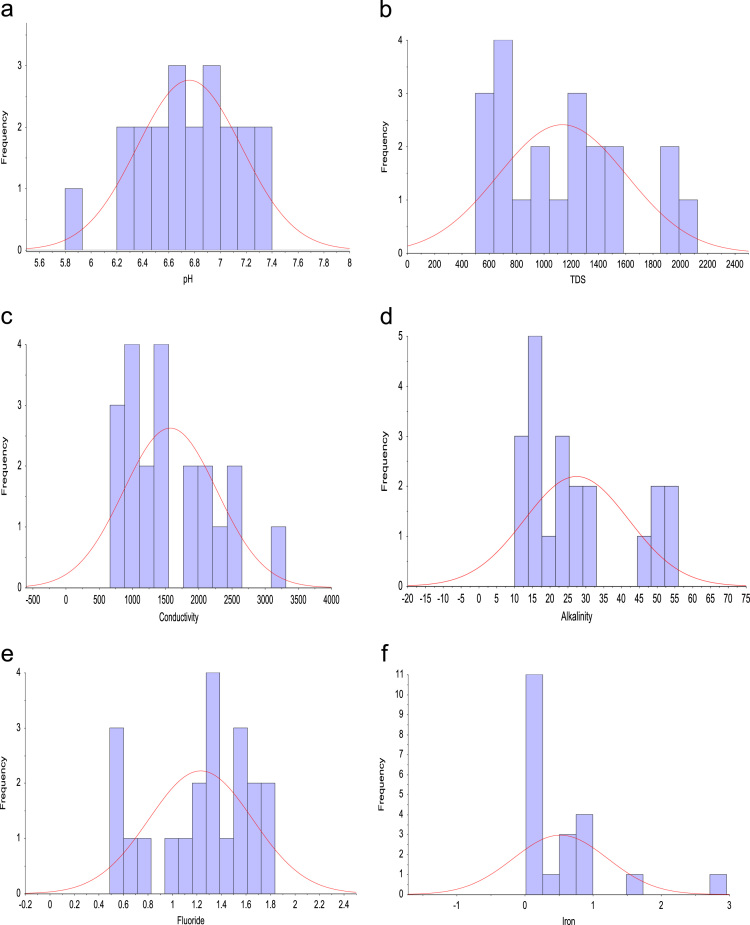

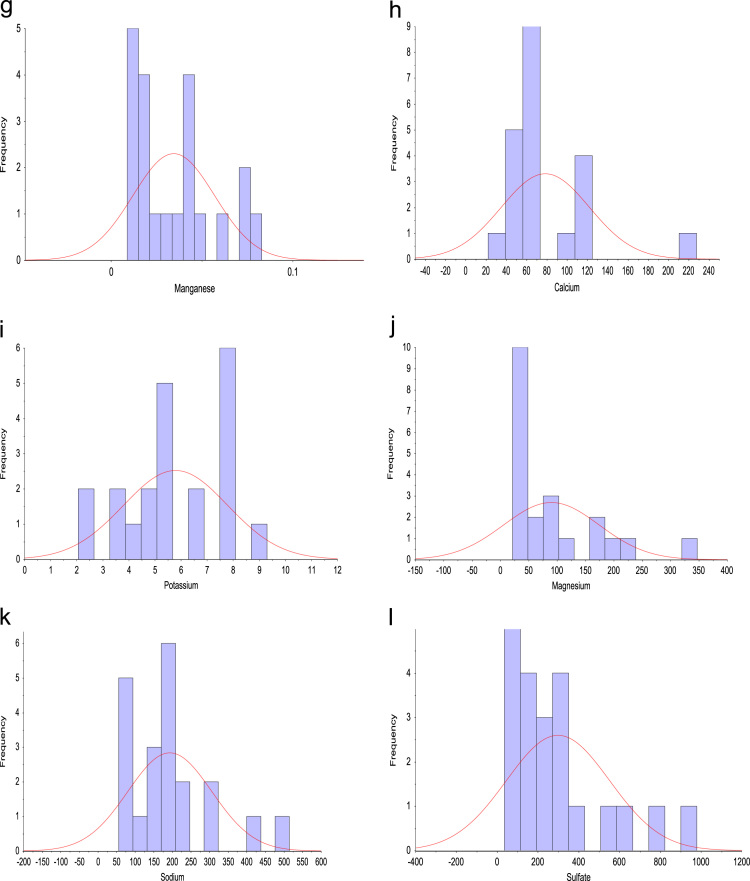

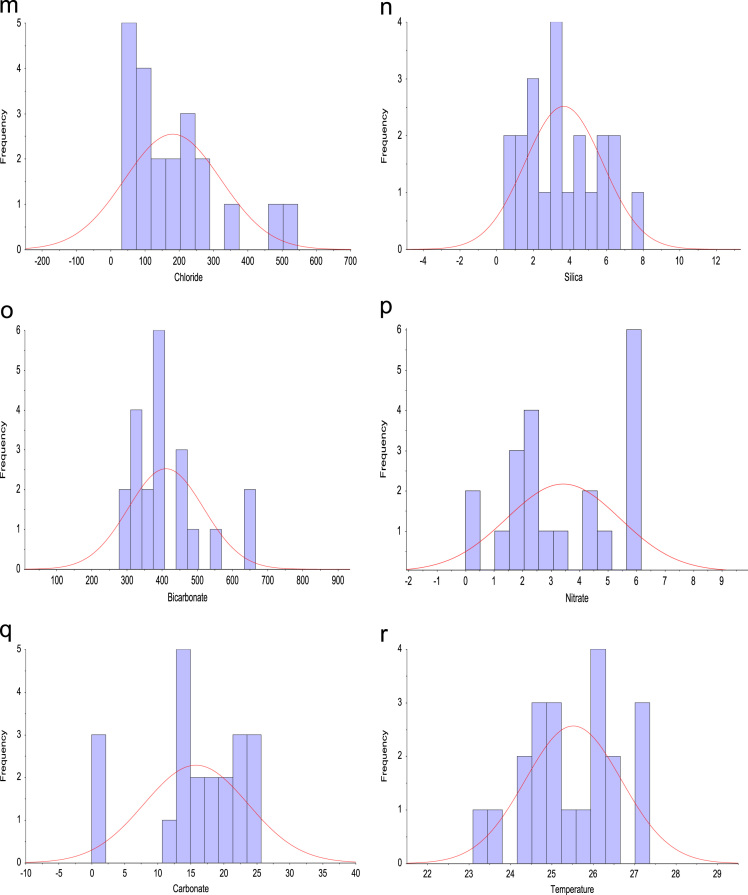
Frequency distribution of (a) pH (b) TDS (c) EC (d) Alkalinity (e) F^–^ (f) Fe^2+^: frequency distribution of (g) Mn (h) Ca^2+^ (i) K^+^ (j) Mg^2+^ (k) Na^+^ (l) ${SO}_{4}^{2-}$: frequency distribution of (m) Cl^–^ (n) (l) SiO_2_ (o) HCO^3–^ (p) NO^3–^ (q) ${CO}_{3}^{2-}$ (r) Temperature.

Standard sampling procedures were adopted throughout the study. The samples collected were stored accordingly, in line with stipulated methods used for water and wastewater before analysis was conducted. On site, sensitive water quality parameters such as pH, Total dissolved solids (TDS), alkalinity, electrical conductivity (EC) and temperature were measured using HANNA – HI2030 Salinity/TDS/EC meter multiparameter and HANNA – HI98130 probe. Iron and manganese concentration was measured with The flame absorption spectrophotometer (FAAS) [5], [6], while the fluoride concentration in the samples was measured with a calibrated potentiometric ion-selection electrode (HANNA–HI5315) and a professional water-resistant portable ORP/pH/ISE meter (HANNA–HI98191). As for the other parameters, their ionic contents were measured using standard analytical method [1]. The descriptive plots of the collected tap water samples are presented in Fig. 2(a–r).

**Fig. 3:**
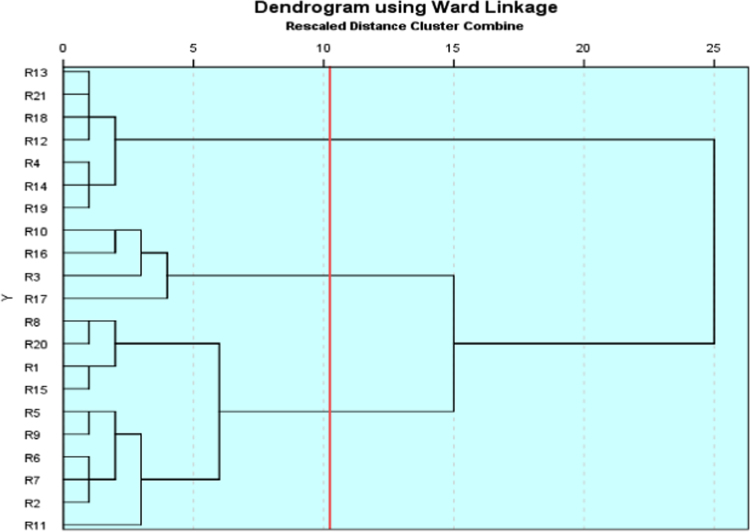
Dendrogram representation of all water samples from different locations.

To consider possible connections and the extent of similarity and distinction existing between the different locations, Hierarchical Cluster Analysis (HCA) technique was adopted. Fig. 3 presents the Ward linkage dendrogram that classified the observed samples.

The important parameters used for calculating the exposure risk associated with fluoride and iron contamination in children and adult, given by the United States Environmental Protection Agency (USEPA) [7] was used to compute the Average daily dose (ADD) of each contaminant. The parameters were inserted in Eqs. (1), (2) to evaluate the exposure risk associated with fluoride and iron concentration considering ingestion (*ADD_IN_*) [8], [9], [10], [11] and dermal (*ADD_DE_*) pathways respectively.

(1) $$ADD_{IN}=\frac{C_{wp}\times IR_{w}\times EF_{r}\times ED}{BW\times AT_{r}}$$

(2) $$ADD_{DE}=\frac{C_{wp}\times SA\times K_{p}\times EF_{r}\times ED\times ET\times CF}{BW\times AT_{r}}$$

The data in Table 1, Table 2 presents the *ADD_IN_* and *HQ_IN_* of Iron and fluoride concentration in the water samples respectively. It also considers the ADD for adult and child obtained from different locations (R1−R21). In addition, the data in Table 3, Table 4 presents the *ADD_DE_* and *HQ_DE_* of Iron and fluoride respectively. The values obtained from Table 1, Table 2, Table 3, Table 4, were used to calculate the total Hazard Index (*HI*) values (Table 5) for iron and fluoride. The total Hazard index ($HI_{total}$) was determined for non-carcinogenic risk according to Eq. (3);

(3) $$\begin{array}{c} HI_{total}=\frac{ADD_{IN}}{RfD}+\frac{ADD_{DE}}{RfD}\\ HI_{total}=HQ_{IN}+HQ_{DE} \end{array}$$

where $C_{wp}$ is concentration of trace element; $IR_{w}$ is water ingestion rate (taken as 1 L/ay and 2 L/day for child and adult respectively) US EPA 2011; ED is the exposure duration (taken as 6 years and 30 years for child and adult respectively); $EF_{r}$ is the exposure frequency (taken as 365 days); BW means body weight (taken to be 32.5 kg and 72 kg for child and adult respectively) [12], [13], [14], [15]; $AT_{r}$ means average time (taken as 2190 days and 10,950 days for child and adult respectively) [7]; SA represents skin surface area (taken to be 6365 cm^2^ and 19,652 cm^2^ for child and adult respectively); ET means exposure time (taken as 350 days); $K_{p}$ is the skin adherence factor (taken as 0.001); CF represents conversion factor (taken to be 0.001). RfD is the oral reference dose (taken as 60 μg/kg−day for fluoride, according to the Integrated Risk Information System (IRIS) database of the US EPA and [4]. RfD of iron through ingestion and dermal pathways are 300 μg/kg−day and 45 μg/kg−day respectively [15], [16]. A significant risk may occur for non-cancer effect if the Hazard index is greater than one. The Hazard index value less than one means that there is no chance of non-cancer effect happening [17], [18], [19], [20], [21].

**Table 1:** *ADD_IN_* and *HQ_IN_* values via ingestion pathway for iron.

**Sample points**	***ADD**_**IN**_***(Adults)**	***ADD**_**IN**_***(Children)**	***HQ**_**IN**_***(Adults)**	***HQ**_**IN**_***(Children)**
R1	12.778±1.735	14.067±1.91	4.259E−02	4.689E−02
R2	6.759±0.699	7.441±0.77	2.253E−02	2.480E−02
R3	19.907±0.16	21.916±0.177	6.636E−02	7.305E−02
R4	24.352±2.362	26.809±2.601	8.117E−02	8.936E−02
R5	82.222±1.944	90.520±2.141	2.741E−01	3.017E−01
R6	0.935±0.439	1.030±0.484	3.117E−03	3.432E−03
R7	0.454±0.158	0.499±0.174	1.512E−03	1.665E−03
R8	20.926±1.807	23.038±1.99	6.975E−02	7.679E−02
R9	23.611±1.822	25.994±2.005	7.870E−02	8.665E−02
R10	0.593±0.294	0.652±0.324	1.975E−03	2.175E−03
R11	2.167±0.530	2.385±0.583	7.222E−03	7.951E−03
R12	23.278±0.583	25.627±1.278	7.759E−02	8.542E−02
R13	16.241±1.115	17.880±1.228	5.414E−02	5.960E−02
R14	1.176±0.116	1.295±0.127	3.920E−03	4.315E−03
R15	5.648±0.331	6.218±0.365	1.883E−02	2.073E−02
R16	1.296±0.163	1.427±0.179	4.321E−03	4.757E−03
R17	41.944±1.273	46.177±1.401	1.398E−01	1.539E−01
R18	0.787±0.212	0.866±0.234	2.623E−03	2.888E−03
R19	0.824±0.016	0.907±0.018	2.747E−03	3.024E−03
R20	2.991±0.070	3.293±0.077	9.969E−03	1.098E−02
R21	14.907±0.160	16.412±0.177	4.969E−02	5.471E−02

**Table 2:** *ADD_IN_* and *HQ_IN_* values via ingestion pathway for fluoride.

**Sample points**	***ADD**_**IN**_***(Adults)**	***ADD**_**IN**_***(Children)**	***HQ**_**IN**_***(Adults)**	***HQ**_**IN**_***(Children)**
R1	28.472±0.139	31.346±0.153	4.745E−01	5.224E−01
R2	36.481±0.424	40.163±0.467	6.080E−01	6.694E−01
R3	42.963±0.424	47.299±0.467	7.160E−01	7.883E−01
R4	45.741±2.837	50.357±3.124	7.623E−01	8.393E−01
R5	30.648±3.047	33.741±3.355	5.108E−01	5.624E−01
R6	44.074±8.486	48.522±9.343	7.346E−01	8.087E−01
R7	35.926±5.776	39.551±6.359	5.988E−01	6.592E−01
R8	18.426±2.427	20.285±2.672	3.071E−01	3.381E−01
R9	13.843±1.995	15.240±2.196	2.307E−01	2.540E−01
R10	40.185±11.008	44.241±12.118	6.698E−01	7.373E−01
R11	22.500±3.889	24.771±4.281	3.750E−01	4.128E−01
R12	13.796±2.625	15.189±2.890	2.299E−01	2.531E−01
R13	37.500±3.758	41.284±4.137	6.250E−01	6.881E−01
R14	34.259±16.966	37.717±18.678	5.710E−01	6.286E−01
R15	46.019±1.580	50.663±1.739	7.670E−01	8.444E−01
R16	51.019±20.385	56.167±22.442	8.503E−01	9.361E−01
R17	48.796±0.893	53.721±0.983	8.133E−01	8.953E−01
R18	36.852±10.047	40.571±11.061	6.142E−01	6.762E−01
R19	34.815±0.160	38.328±0.177	5.802E−01	6.388E−01
R20	42.685±0.160	46.993±0.177	7.114E−01	7.832E−01
R21	13.611±0.278	14.985±0.306	2.269E−01	2.497E−01

**Table 3:** *ADD_DE_* and *HQ_DE_* values for iron.

**Sample points**	***ADD**_**DE**_***(Adults)**	***ADD**_**DE**_***(Children)**	***HQ**_**DE**_***(Adults)**	***HQ**_**DE**_***(Children)**
R1	0.085±1.160E−02	0.050±6.761E−03	1.900E−03	1.107E−03
R2	0.045±4.677E−03	0.026±2.724E−03	1.005E−03	5.854E−04
R3	0.133±1.073E−03	0.078±6.250E−04	2.959E−03	1.724E−03
R4	0.163±1.580E−02	0.095±9.207E−03	3.620E−03	2.109E−03
R5	0.550±1.301E−02	0.320±7.578E−03	1.222E−02	7.121E−03
R6	0.006±2.940E−03	0.004±1.713E−03	1.390E−04	8.099E−05
R7	0.003±1.057E−03	0.002±6.156E−04	6.745E−05	3.929E−05
R8	0.140±1.209E−02	0.082±7.044E−03	3.111E−03	1.812E−03
R9	0.158±1.219E−02	0.092±7.099E−03	3.510E−03	2.045E−03
R10	0.004±1.970E−03	0.002±1.147E−03	8.810E−05	5.132E−05
R11	0.014±3.545E−03	0.008±2.065E−03	3.221E−04	1.876E−04
R12	0.156±7.767E−03	0.091±4.525E−03	3.461E−03	2.016E−03
R13	0.109±7.462E−03	0.063±4.347E−03	2.414E−03	1.407E−03
R14	0.008±7.737E−04	0.005±4.507E−04	1.748E−04	1.018E−04
R15	0.038±2.217E−03	0.022±1.292E−03	8.397E−04	4.892E−04
R16	0.009±1.089E−03	0.005±6.343E−04	1.927E−04	1.123E−04
R17	0.281±8.516E−03	0.163±4.961E−03	6.236E−03	3.633E−03
R18	0.005±1.419E−03	0.003±8.268E−04	1.170E−04	6.816E−05
R19	0.006±1.073E−04	0.003±6.250E−05	1.225E−04	7.137E−05
R20	0.020±4.677E−04	0.012±2.724E−04	4.446E−04	2.590E−04
R21	0.100±1.073E−03	0.058±6.250E−04	2.216E−03	1.291E−03

**Table 4:** *ADD_DE_* and *HQ_DE_* values for fluoride.

**Sample points**	***ADD**_**DE**_***(Adults)**	***ADD**_**DE**_***(Children)**	***HQ**_**DE**_***(Adults)**	***HQ**_**DE**_***(Children)**
R1	0.190±9.29E−04	0.111±5.41E−04	3.88E−03	2.26E−03
R2	0.244±2.84E−03	0.142±1.65E−03	4.97E−03	2.90E−03
R3	0.287±2.84E−03	0.167±1.65E−03	5.85E−03	3.41E−03
R4	0.306±1.90E−02	0.178±1.11E−02	6.21E−03	3.62E−03
R5	0.205±2.04E−02	0.119±1.19E−02	4.22E−03	2.46E−03
R6	0.295±5.68E−02	0.172±3.31E−02	6.10E−03	3.55E−03
R7	0.240±3.86E−02	0.140±2.25E−02	4.95E−03	2.88E−03
R8	0.123±1.62E−02	0.072±9.46E−03	2.52E−03	1.47E−03
R9	0.093±1.34E−02	0.054±7.78E−03	1.86E−03	1.08E−03
R10	0.269±7.36E−02	0.157±4.29E−02	5.61E−03	3.27E−03
R11	0.151±2.60E−02	0.088±1.52E−02	3.01E−03	1.75E−03
R12	0.092±1.76E−02	0.054±1.02E−02	1.86E−03	1.08E−03
R13	0.251±2.51E−02	0.146±1.46E−02	5.16E−03	3.01E−03
R14	0.229±1.14E−01	0.134±6.61E−02	4.43E−03	2.58E−03
R15	0.308±1.06E−02	0.179±6.16E−03	6.28E−03	3.66E−03
R16	0.341±1.36E−01	0.199±7.94E−02	7.19E−03	4.19E−03
R17	0.326±5.97E−03	0.190±3.48E−03	6.66E−03	3.88E−03
R18	0.247±6.72E−02	0.144±3.92E−02	5.17E−03	3.01E−03
R19	0.233±1.07E−03	0.136±6.25E−04	4.74E−03	2.76E−03
R20	0.286±1.07E−03	0.166±6.25E−04	5.82E−03	3.39E−03
R21	0.091±1.86E−03	0.053±1.08E−03	1.86E−03	1.08E−03

**Table 5:** Total Hazard Index (*HI*) for iron and fluoride.

**Sample points**	***HI***_***total***_			
	**Iron**		**Fluoride**	
	Adult	Child	Adult	Child
R1	4.449E−02	4.800E−02	4.784E−01	5.247E−01
R2	2.354E−02	2.539E−02	6.130E−01	6.723E−01
R3	6.932E−02	7.478E−02	7.219E−01	7.917E−01
R4	8.479E−02	9.147E−02	7.686E−01	8.429E−01
R5	2.863E−01	3.089E−01	5.150E−01	5.648E−01
R6	3.256E−03	3.513E−03	7.407E−01	8.123E−01
R7	1.580E−03	1.704E−03	6.037E−01	6.621E−01
R8	7.286E−02	7.860E−02	3.096E−01	3.396E−01
R9	8.221E−02	8.869E−02	2.326E−01	2.551E−01
R10	2.063E−03	2.226E−03	6.754E−01	7.406E−01
R11	7.544E−03	8.139E−03	3.780E−01	4.146E−01
R12	8.105E−02	8.744E−02	2.318E−01	2.542E−01
R13	5.655E−02	6.101E−02	6.302E−01	6.911E−01
R14	4.095E−03	4.417E−03	5.754E−01	6.312E−01
R15	1.967E−02	2.122E−02	7.733E−01	8.480E−01
R16	4.514E−03	4.869E−03	8.575E−01	9.403E−01
R17	1.461E−01	1.576E−01	8.199E−01	8.992E−01
R18	2.740E−03	2.956E−03	6.194E−01	6.792E−01
R19	2.869E−03	3.095E−03	5.850E−01	6.416E−01
R20	1.041E−02	1.123E−02	7.172E−01	7.866E−01
R21	5.191E−02	5.600E−02	2.287E−01	2.508E−01
