# Supplementary material for: Human health risk assessment data of trace elements concentration in tap water—Abeokuta South, Nigeria
Source: Data Brief. 2018 Apr 19;18:1416–26. doi: 10.1016/j.dib.2018.04.041 (PMC5997959; doi:10.1016/j.dib.2018.04.041)
Supplement: Supplementary file 1 — Supplementary material [file mmc1.docx]

EMENIKE CHIDOZIE PRAISEGOD

*Department of Civil Engineering, Covenant University, Ota, Ogun State, Nigeria.*

*Cranfield Water Science Institute, School of Water, Energy and Environment, Cranfield University. Bedfordshire. The United Kingdom.*

Email: [borngreatemenike@gmail.com, praisegod.emenike@covenantuniversity.edu.ng](mailto:borngreatemenike@gmail.com,%20praisegod.emenike@covenantuniversity.edu.ng)

**March 27, 2018**

**Data In Brief**

Dear Editor,

The authors declare no conflict of interest.

*Signatures and names of all the authors*

**Emenike P.C**

**Corresponding Author on behalf of co-authors:**

**TENEBE I.T.**

**KAFI C.O.**
